# Supplementary material for: The association between ADIPOQ gene variants (rs266729, rs2241766, rs1501299) and acute myocardial infarction in Vietnamese patients with type 2 diabetes mellitus
Source: PeerJ. 2025 Oct 3;13:e20145. doi: 10.7717/peerj.20145 (PMC12499558; doi:10.7717/peerj.20145)
Supplement: Supplemental Information 1 [file peerj-13-20145-s001.docx]

**DATA DICTIONARY**

| **Variable name** | **Variable values** | **Variable label** |
| --- | --- | --- |
| **code** | Text | Patient Code |
| **group** | 0=Control; 1=Case; | Group |
| **age** | Numeric (N = 550; from: 31 to 94) | Age (year) |
| **gender** | 0=Female; 1=Male; | Gender |
| **duration** | Numeric (N = 550; from: 0 to 35) | Duration of T2DM |
| **dm** | 0=No; 1=Yes; | Family history of diabetes |
| **hyper** | 0=No; 1=Yes; | Hypertension |
| **dys** | 0=No; 1=Yes; | Dyslipidemia |
| **stroke** | 0=No; 1=Yes; | History of stroke |
| **pad** | 0=No; 1=Yes; | History of peripheral artery disease |
| **smoke** | 0=No; 1=Yes; | Smoking status |
| **physical** | 0=No; 1=Yes; | Physical activity |
| **bmi** | Numeric (N = 550; from: 15.63 to 35.16) | Body Mass Index (kg/m2) |
| **waist** | Numeric (N = 550; from: 62 to 130) | Waist circumference (cm) |
| **hip** | Numeric (N = 550; from: 63 to 143) | Hip circumference (cm) |
| **whr** | Numeric (N = 550; from: .76 to 1.24) | Waist-to-hip ratio |
| **systolic** | Numeric (N = 550; from: 82 to 213) | Systolic blood pressure (mmHg) |
| **diastolic** | Numeric (N = 550; from: 39 to 113) | Diastolic blood pressure (mmHg) |
| **glucose** | Numeric (N = 550; from: 56 to 555) | Glucose (mg/dL) |
| **hba1c** | Numeric (N = 548; from: 5.2 to 14.9) | HbA1c (%) |
| **chol** | Numeric (N = 493; from: 22 to 458) | Total cholesterol (mg/dL) |
| **hdl** | Numeric (N = 492; from: 7 to 82) | HDL-C (mg/dL) |
| **ldl** | Numeric (N = 547; from: 23 to 289) | LDL-C (mg/dL) |
| **trig** | Numeric (N = 547; from: 49 to 1342) | Triglycerides (mg/dL) |
| **cre** | Numeric (N = 550; from: .45 to 10.8) | Creatinine (mg/dL) |
| **egfr** | Numeric (N = 550; from: 4 to 149) | eGFR (mL/min/1.73m2) |
| **ef** | Numeric (N = 513; from: 15 to 87) | Ejection fraction (%) |
| **su** | 0=No; 1=Yes; | Antidiabetic medications - Sulfonylureas |
| **met** | 0=No; 1=Yes; | Antidiabetic medications - Metformin |
| **dpp4i** | 0=No; 1=Yes; | Antidiabetic medications - DPP-4 inhibitors |
| **sglt2i** | 0=No; 1=Yes; | Antidiabetic medications - SGLT2 inhibitors |
| **glp1** | 0=No; 1=Yes; | Antidiabetic medications - GLP-1 analogs |
| **insulin** | 0=No; 1=Yes; | Antidiabetic medications - Insulin |
| **hypermed** | 0=No; 1=Yes; | Antihypertensive medications |
| **arb** | 0=No; 1=Yes; | Antihypertensive medications - ARB |
| **acei** | 0=No; 1=Yes; | Antihypertensive medications - ACE inhibitors |
| **ccb** | 0=No; 1=Yes; | Antihypertensive medications - CCB |
| **bb** | 0=No; 1=Yes; | Antihypertensive medications - Beta-blockers |
| **diu** | 0=No; 1=Yes; | Antihypertensive medications - Diuretics |
| **asa** | 0=No; 1=Yes; | Aspirin use |
| **fibrate** | 0=No; 1=Yes; | Fibrate use |
| **statin** | 0=No; 1=Yes; | Statin use |
| **rs266729** | 1=C/C; 2=C/G; 3=G/G; | rs266729 (C/C C/G G/G) |
| **rs2241766** | 1=T/T; 2=T/G; 3=G/G; | rs2241766 (T/T T/G G/G) |
| **rs1501299** | 1=G/G; 2=G/T; 3=T/T; | rs1501299 (G/G G/T T/T) |
