# Supplementary material for: The association between ADIPOQ gene variants (rs266729, rs2241766, rs1501299) and acute myocardial infarction in Vietnamese patients with type 2 diabetes mellitus
Source: PeerJ. 2025 Oct 3;13:e20145. doi: 10.7717/peerj.20145 (PMC12499558; doi:10.7717/peerj.20145)
Supplement: Supplemental Information 4 [file peerj-13-20145-s004.docx]

Supplemental Table S1: Hardy-Weinberg Equilibrium Analysis of *ADIPOQ* SNPs

|  | **Genotype** | | | **Allele** | | **P-value (HWE)** |
| --- | --- | --- | --- | --- | --- | --- |
| **SNP rs266729** | **C/C** | **C/G** | **G/G** | **C** | **G** |  |
| Total (n=550) | 290 | 217 | 43 | 797 | 303 | **0.83** |
| Cases (n=275) | 150 | 111 | 14 | 411 | 139 | **0.34** |
| Controls (n=275) | 140 | 106 | 29 | 386 | 164 | **0.20** |
| **SNP rs2241766** | **T/T** | **T/G** | **G/G** | **T** | **G** |  |
| Total (n=550) | 289 | 205 | 56 | 783 | 317 | **0.037** |
| Cases (n=275) | 123 | 124 | 28 | 370 | 180 | **0.78** |
| Controls (n=275) | 166 | 81 | 28 | 413 | 137 | **0.00063** |
| **SNP 1501299** | **G/G** | **G/T** | **T/T** | **G** | **T** |  |
| Total (n=550) | 313 | 198 | 39 | 824 | 276 | **0.31** |
| Cases (n=275) | 165 | 97 | 13 | 427 | 123 | **0.86** |
| Controls (n=275) | 148 | 101 | 26 | 397 | 153 | **0.18** |
